# Imaging Characteristics of Tongue Hematoma and Pseudoaneurysm Following Tooth Extraction Requiring Emergency Liquid Embolization

**DOI:** 10.7759/cureus.39731

**Published:** 2023-05-30

**Authors:** Farris Ahmed, Manisha Koneru, Rahul Garg, Hamza Shaikh

**Affiliations:** 1 Radiology, Rowan-Virtua School of Osteopathic Medicine, Stratford, USA; 2 Radiology, Cooper Medical School of Rowan University, Camden, USA; 3 Radiology, Cooper University Hospital, Camden, USA; 4 Neurosurgery, Cooper University Hospital, Camden, USA

**Keywords:** liquid embolization, lingual artery, tooth extraction, pseudoaneurysm, artery pseudoaneurysm

## Abstract

Our case describes the imaging characteristics of a tongue hematoma and lingual artery pseudoaneurysm following oral surgery, treated with a liquid embolic agent prior to repeat instrumentation. Identifying particular imaging cues that suggest underlying vascular pathology is essential to prevent unnecessary, potentially fatal instrumentation. A liquid embolizing agent can be used to endovascularly treat an unstable pseudoaneurysm in the oral cavity.

## Introduction

Pseudoaneurysms, or “false aneurysms,” are rare lesions in the vasculature [1]. They occur in between the tunica media and tunica adventitia vessel wall layers, which compromises wall integrity [2]. Unlike true aneurysms, the asymmetric and intralayer positioning of pseudoaneurysms increases their risk of rupture. The incidence of pseudoaneurysms in the head and neck is unknown due to the limited prior literature on the topic. Pseudoaneurysms occur following trauma or force and are sometimes secondary to surgical procedures [3]. Due to their relative instability, pseudoaneurysms can cause sudden, extensive hemorrhage postoperatively [4]. Consequently, pseudoaneurysms require rapid identification on imaging studies and subsequent close monitoring or emergent treatment to address potentially fatal hemorrhage.

Pseudoaneurysms can occur anywhere in the vasculature. Prior approaches for aneurysm and pseudoaneurysm treatment include vascular plugs, platinum coiling, mechanical embolization, and surgical repair [1,3-5]. However, novel approaches capitalizing on current advancements in interventional techniques show potential for use in pseudoaneurysm treatment.

We describe imaging findings of a lingual artery pseudoaneurysm and tongue hematoma on computed tomography (CT) and magnetic resonance imaging (MRI) following oral surgery treated minimally invasively using the Onyx 18 liquid embolization agent (Medtronic, Minneapolis, MN, USA). 

This case was previously presented as a meeting poster at the 2023 American Society of Neuroradiology Annual Meeting on April 29, 2023-May 3, 2023.

## Case presentation

A 61-year-old female patient with a history of atrial fibrillation on oral anticoagulation, hypertension, hyperlipidemia, recent drug-induced liver injury, and gastroesophageal reflux disease underwent a tooth extraction at an external oral surgery office. One week afterward, she reported left-sided tongue pain and swelling that progressively worsened, prompting a presentation to the emergency department per advice from her oral surgeon. 

**Figure 1:**
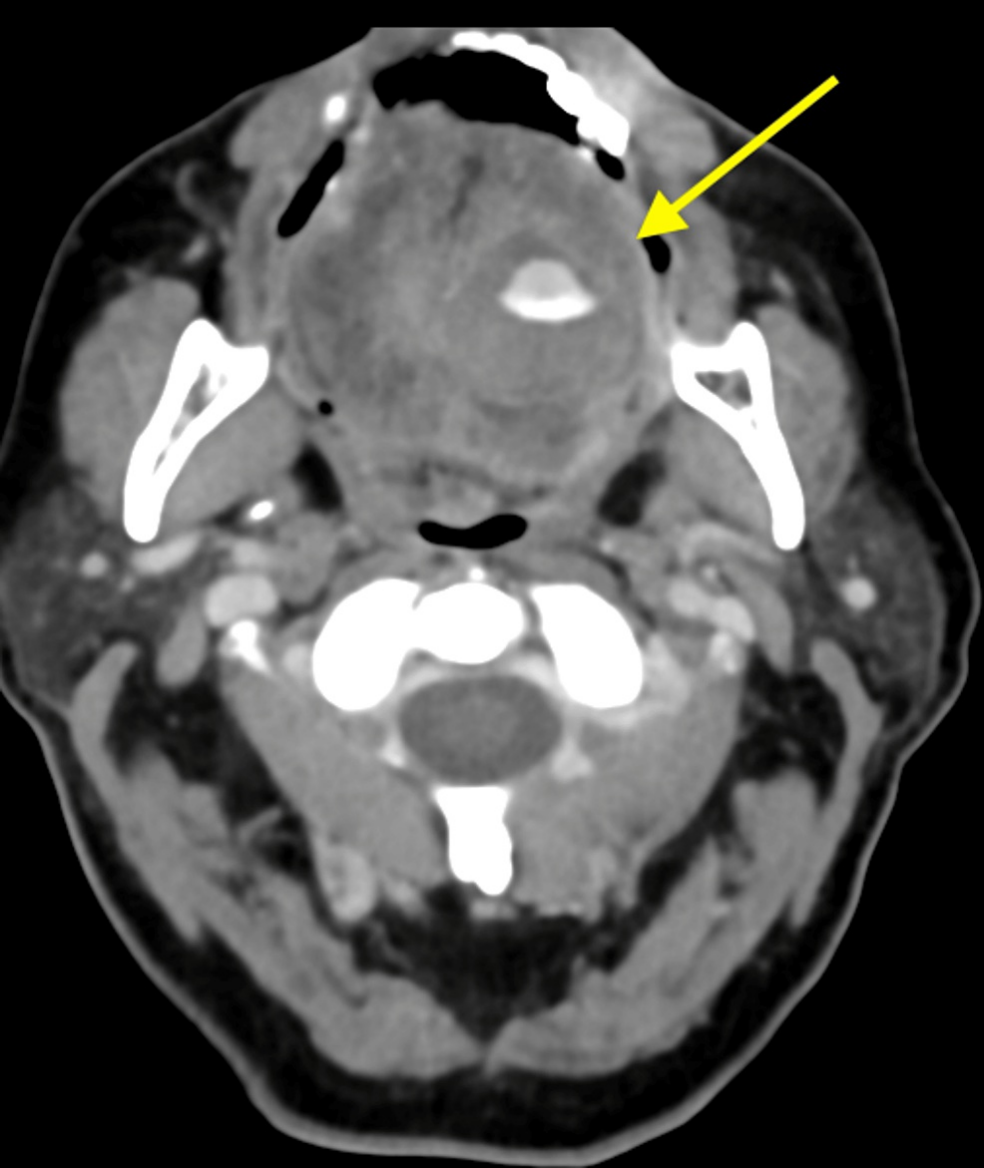
Axial head non-contrast computed tomography demonstrating ventral tongue lesion (arrow). The lesion has a similar appearance to other vascular structures in the same viewing window (e.g., carotid arteries and internal jugular veins).

She had computed tomography (CT) and magnetic resonance imaging (MRI) of the neck. A CT was done on presentation, and an MRI was done 15 hours later, while the patient was being observed. CT with contrast of her neck demonstrated a 2.5 cm lesion on the ventral surface of the left side of the tongue, consistent with a vascular appearance (Figure 1). The lesion demonstrates avid enhancement similar to other vascular structures, specifically the carotid arteries and internal jugular veins. It also showed the extracted left mandibular molar tooth with mild left submandibular space inflammatory changes. At this point, the differential diagnosis included a vascular lesion, an infectious fluid collection, or a mass-like a tumor, with a vascular lesion being at the top of the differential. 

**Figure 2:**
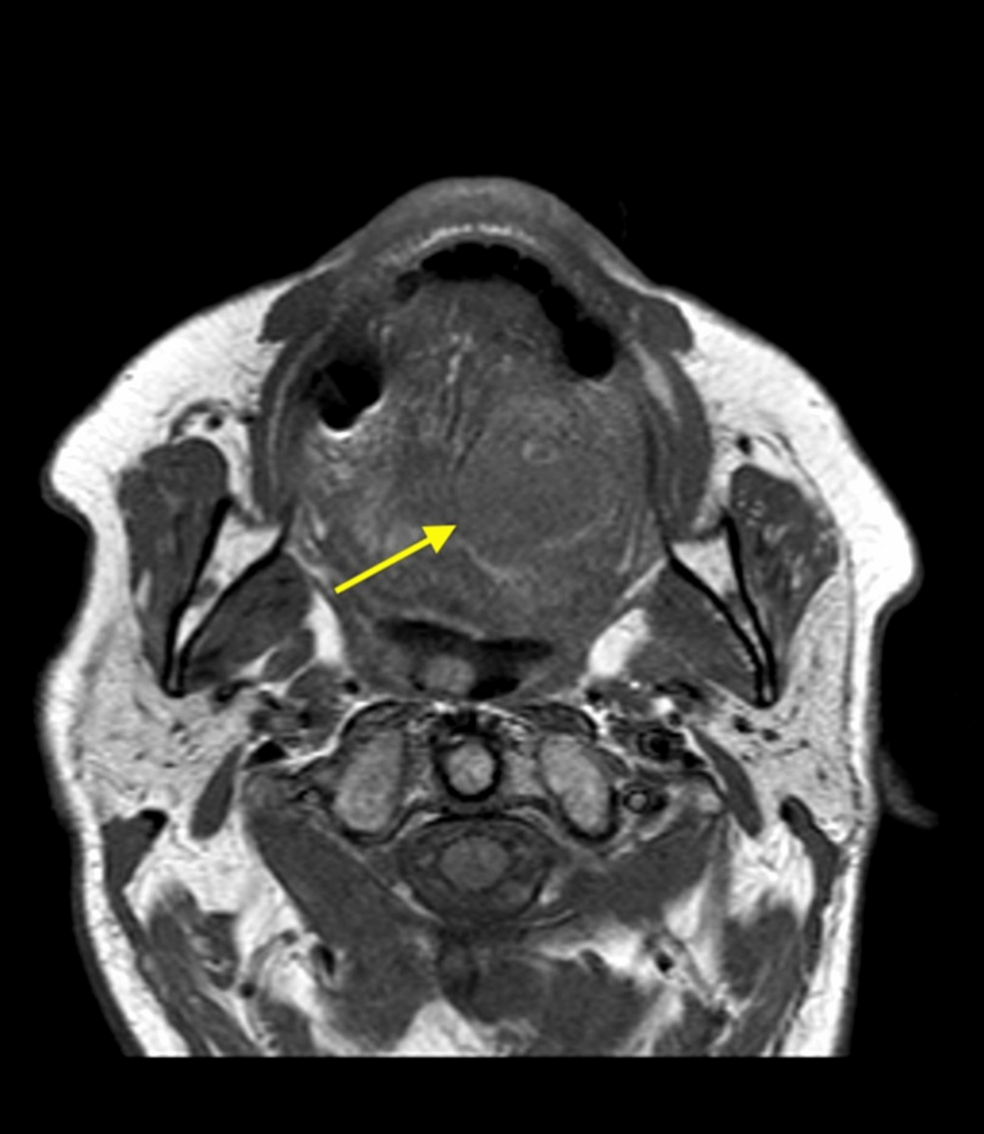
Axial T1-weighted pre-contrast magnetic resonance image of the neck showing an isointense 2.7 cm ovoid lesion within the tongue (arrow).

**Figure 3:**
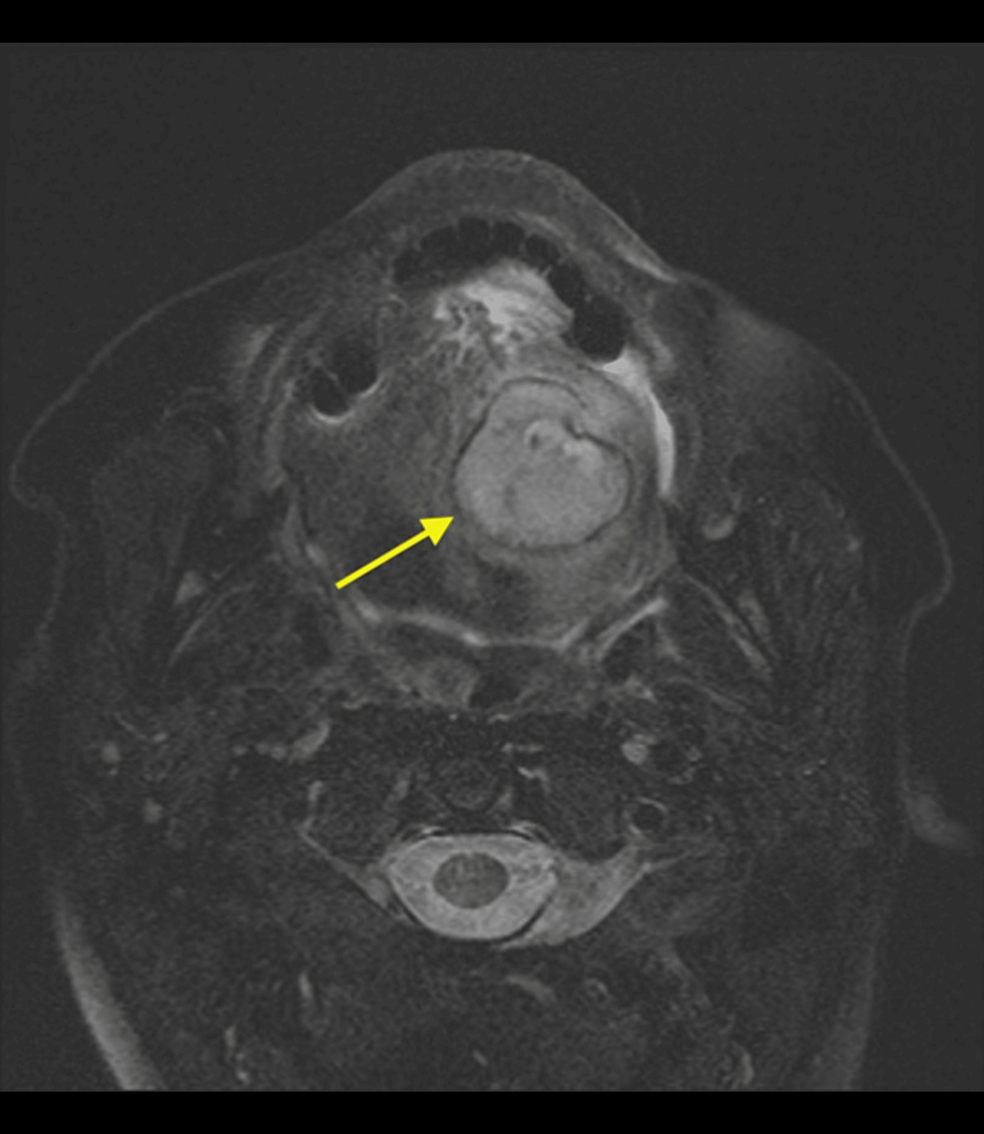
Axial fat-suppressed T2-weighted magnetic resonance image of the neck showing T2 hyperintense lesion with a thin T2 hypointense rim (arrow).

**Figure 4:**
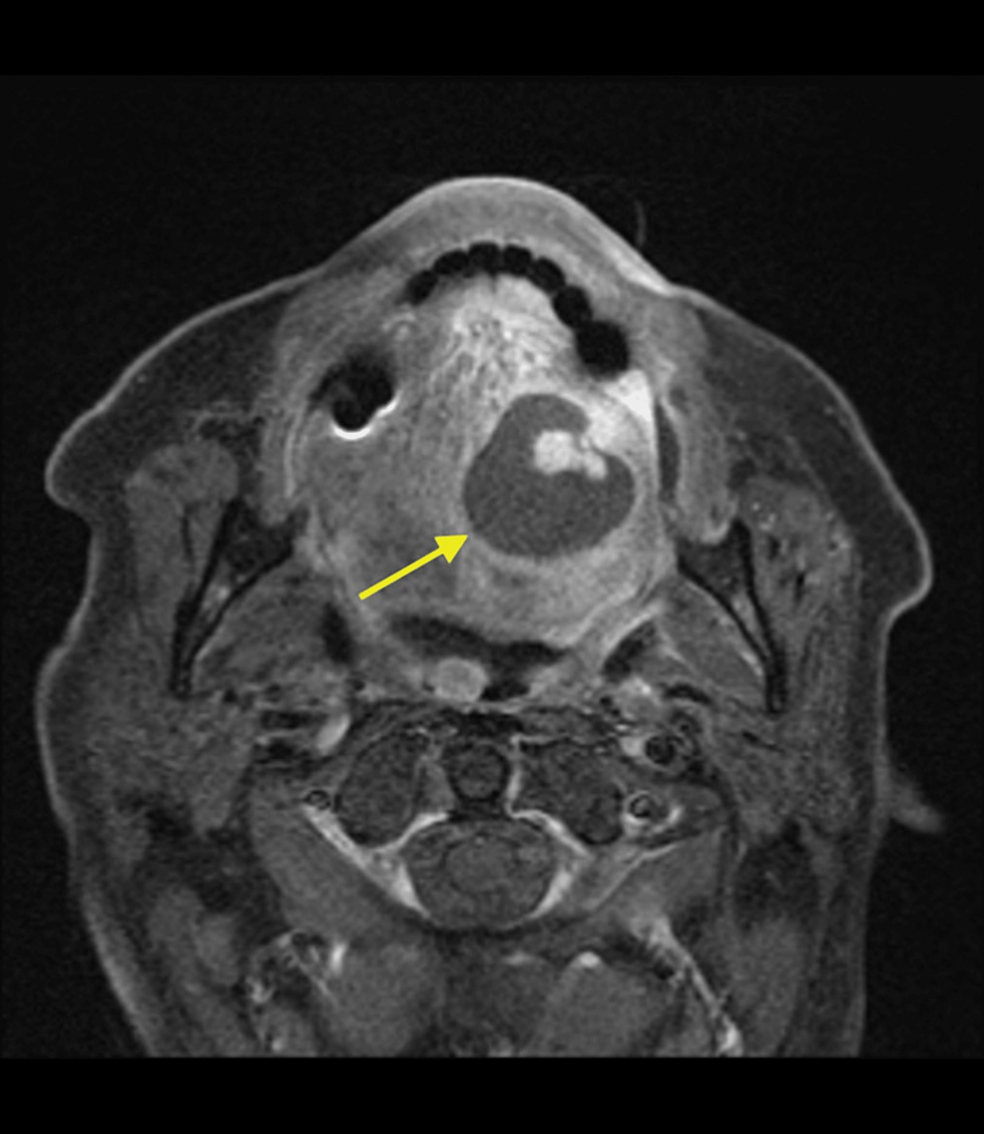
Axial postcontrast fat-suppressed T1-weighted magnetic resonance image of the neck showing avid 1.2 cm central nodular enhancement within the lesion with surrounding fluid hypoenhancement (arrow), most consistent with a lingual artery pseudoaneurysm with surrounding hematoma.

Follow-up MRI with gadolinium contrast of the facial bones was performed. The 2.7 × 2.7 cm ovoid-shaped lesion is isointense on the precontrast T1-weighted axial sequence (Figure 2). The axial T2-weighted fat-suppressed sequence demonstrates an intrinsic T2 hyperintense signal within the lesion and a peripheral thin rim of T2 hypointensity (Figure 3). On the postcontrast, fat-suppressed, T1-weighted sequence, the lesion demonstrates central nodular enhancement measuring 1.2 cm with surrounding hypoenhancing fluid signal intensity, most consistent with a contained vascular bleed in the oral cavity (Figure 4). In total, these findings are consistent with a pseudoaneurysm with surrounding hematomas (Figures 2-4).

**Figure 5:**
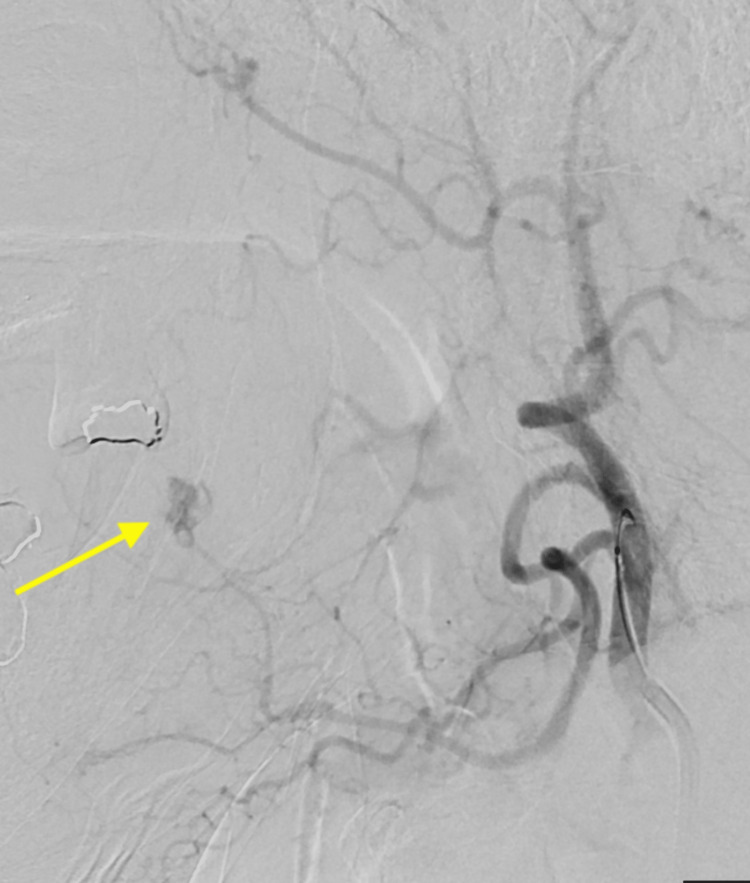
Digital subtraction angiography demonstrating pseudoaneurysm in the lingual artery (arrow).

The immediate concern was that the bleeding near the tongue could obstruct the airway and potentially cause death, prompting urgent intervention. Confirmation of vascular pathology and worsening clinical status prompted the pursuit of endovascular intervention over further imaging like computed tomography angiography (CTA) and magnetic resonance angiography (MRA). The patient was taken to the neuroangiography suite. Using a 0.038” Glidewire guide wire (Terumo Medical Corporation, Somerset, NJ, USA), a 6 French Envoy guiding catheter (Johnson and Johnson Health Care Systems Inc., Piscataway, NJ, USA) was positioned into the left external carotid artery. A Synchro-2 microwire (Stryker, Kalamazoo, MI, USA) and Excelsior SL-10 microcatheter (Stryker, Kalamazoo, MI, USA) were advanced into the lingual branch of the left external carotid artery. Contrast injection in the artery visualized with digital subtraction angiography (DSA) demonstrated a pseudoaneurysm originating from the distal lingual artery (Figure 5). 

**Figure 6:**
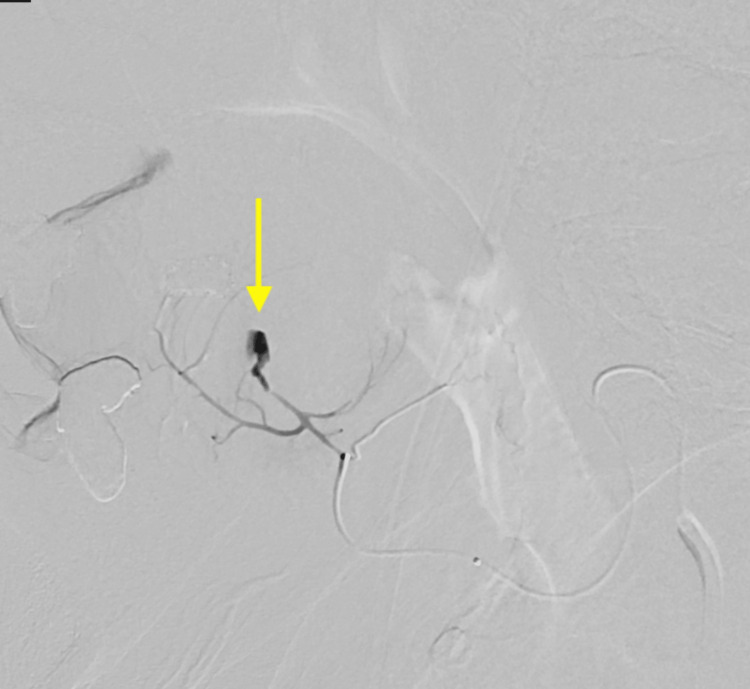
Digital subtraction angiography with selective catheterization of the left lingual artery for Onyx embolization of a pseudoaneurysm (arrow).

**Figure 7:**
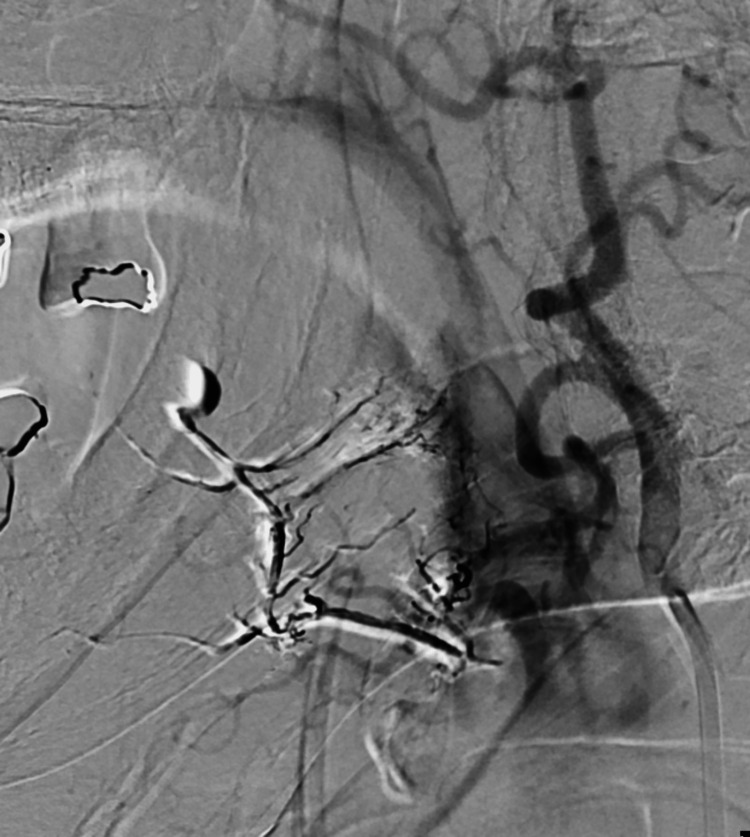
Digital subtraction angiography post-embolization demonstrating successful liquid embolization of the left lingual artery with some residual retained contrast.

At this point, the decision was made to pursue treatment of the aneurysm to prevent further hematoma expansion. After injecting dimethyl sulfoxide (DMSO), Onyx 18 liquid embolizing agent (Medtronic, Minneapolis, MN, USA) was injected at the target site utilizing standard technique (Figure 6). Repeat angiography demonstrated occlusion of the area containing the lingual artery pseudoaneurysm (Figure 7). 

**Figure 8:**
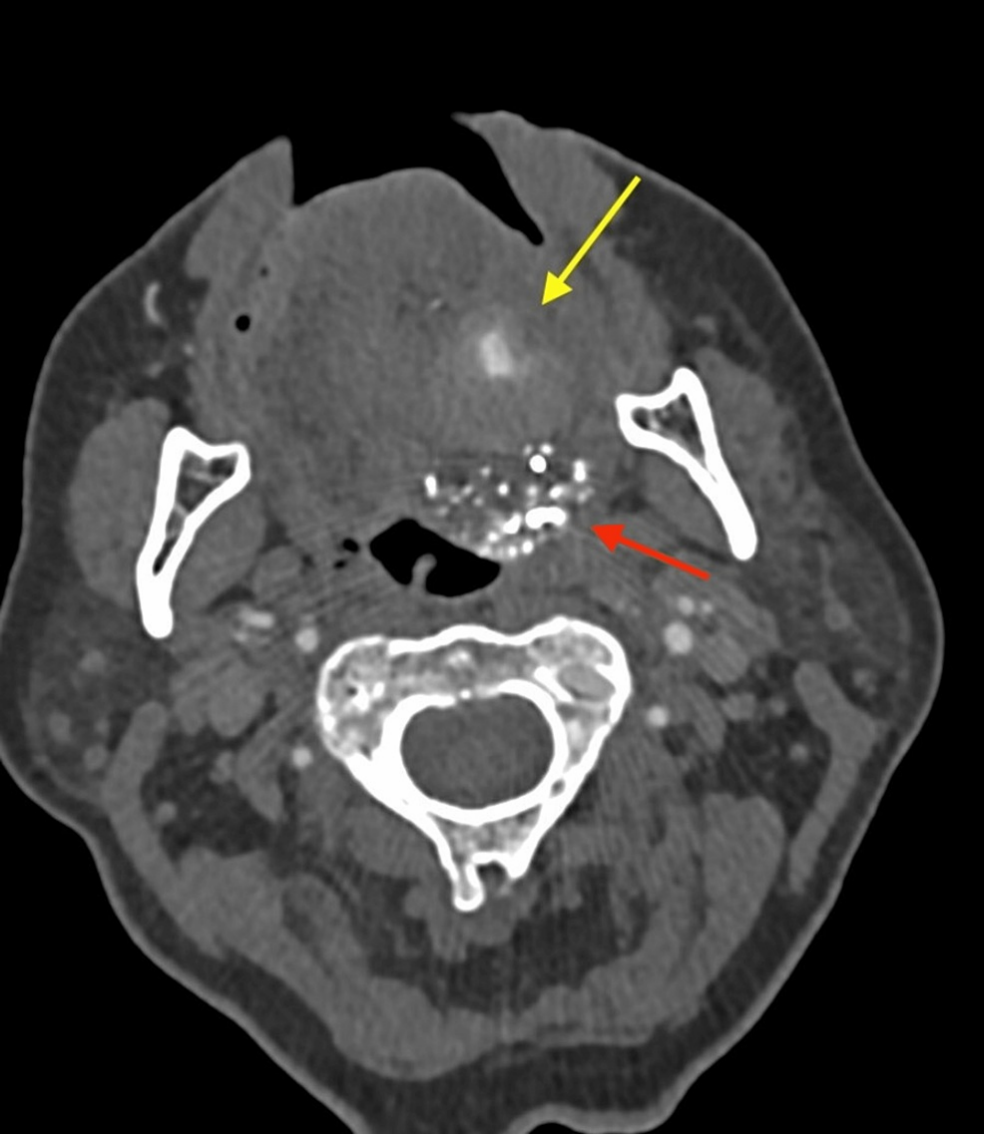
Axial computed tomographic angiography image one-month following endovascular embolization demonstrating a small, ovoid lesion with a residual surrounding hematoma outline (yellow arrow) concerning for potentially residual pseudoaneurysm. Hyperdensity present posterior to the lesion (red arrow) is the embolization material.

**Figure 9:**
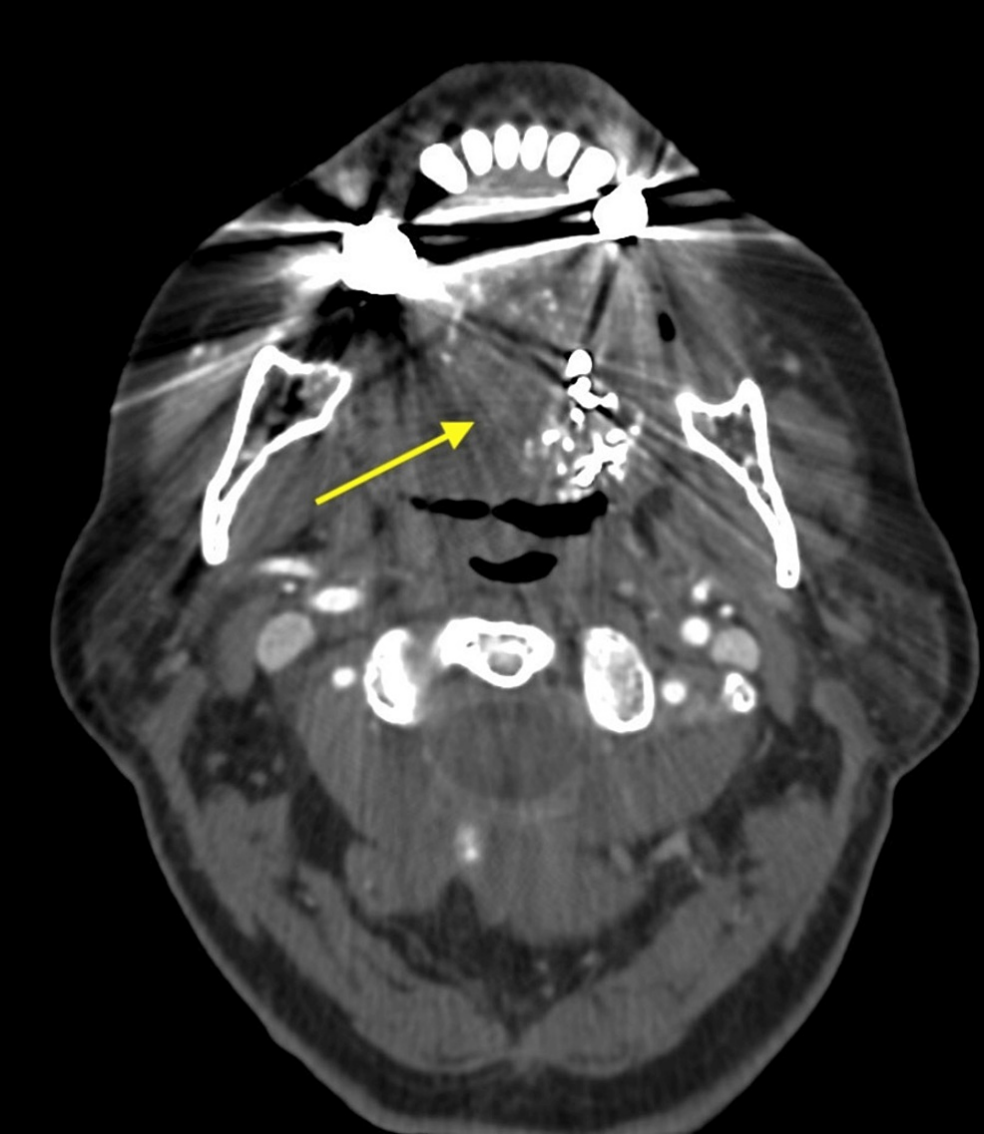
Axial computed tomographic angiography image one-month following endovascular embolization showing hyperdensity from embolization material (arrow) without evidence of either residual pseudoaneurysm or hematoma.

**Figure 10:**
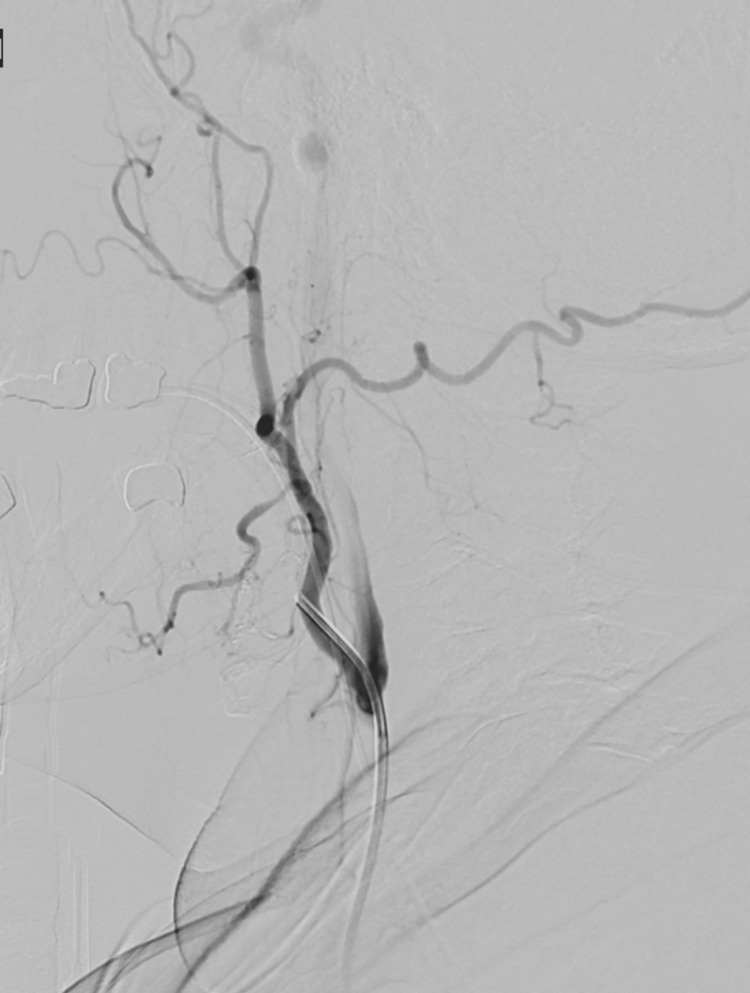
Digital subtraction angiography two months after liquid embolization demonstrating complete resolution of the lingual artery pseudoaneurysm without residual contrast retention.

There were no intraoperative complications, and the postoperative course was uneventful. The bulging mass in the patient’s tongue subsided over a few days. Although residual contrast was present on immediate postoperative imaging, follow-up CT after two weeks showed no retained contrast. CTA performed one-month following endovascular embolization was potentially concerning for a small residual pseudoaneurysm with surrounding hematoma (Figures 8, 9). One month after CTA, a repeat DSA was performed due to concern for residual pseudoaneurysms. The DSA with contrast injected into the external carotid artery demonstrated neither extravasation nor retention of contrast and showed complete resolution of the lingual artery pseudoaneurysm without any further complications (Figure 10).

## Discussion

Approaches to pseudoaneurysms can vary depending on their severity, size, and location. While some relatively stable pseudoaneurysms do not require emergent treatment, other pseudoaneurysms in critical locations require immediate intervention. Pseudoaneurysms in the oral cavity are a particular danger due to the risk of massive bleeding [1]. In this case, the pseudoaneurysm occurred secondary to tooth extraction, but it can occur after other oral surgeries, trauma, or due to tumor invasion [1]. 

Delayed identification of pseudoaneurysms can present with severe complications, particularly if presenting as a ruptured lesion in the head and neck region. Thus, the risk of fatality elucidates the need to promptly and astutely identify pseudoaneurysms on imaging. The imaging findings from the CT and MRI supported a vascular lesion rather than an infection or a mass. It was important to differentiate between a vascular lesion, an infection, or a mass-like tumor early in the diagnostic process. If it were a mass or infection, then the next diagnostic steps would have involved a biopsy, sampling, or drainage of the lesion, which could have provoked bleeding from the fragile pseudoaneurysm and subsequently caused fatal airway obstruction. 

The risk for fatal obstructing hemorrhage illustrates the significance of identifying efficient modalities for pseudoaneurysm management. The literature addressing pseudoaneurysm management is limited. Treatments of pseudoaneurysms in the groin, gastrointestinal system, or aorta can include ultrasound-guided thrombin injections [6]. However, pseudoaneurysms of the neurovasculature are smaller in size than those occurring at other anatomic locations [7]. Prior reported cases of lingual artery pseudoaneurysms developed following oral cavity procedures reported treatment with either surgical intervention or the use of platinum coiling [1,3-5], with one of the cases reporting some degree of embolization [5]. 

Unlike prior described cases, our case utilized Onyx 18, a liquid embolizing agent. Onyx 18 has been used as a preoperative embolizing agent before the use of a plug or pushable coil during surgery [8]. This current case demonstrates an expanded application for Onyx 18 as a primary agent for emergent endovascular pseudoaneurysm treatment without the need for coiling.

## Conclusions

Pseudoaneurysms in the lingual artery occur following oral cavity procedures, emphasizing the importance of rapid evaluation and diagnosis of pseudoaneurysms and associated hematomas as they are potentially fatal postoperative complications. Imaging findings had features more consistent with a vascular lesion, rather than an infectious fluid collection or solid tumor. The workup for the other differential diagnoses would have involved invasive procedures, such as a biopsy, that could have provoked bleeding and fatally obstruct the airway. Consequently, identifying the vascular nature of the lesion on imaging is significant. 

For urgent tamponading of an unstable vascular lesion, Onyx 18 liquid embolization is a useful endovascular, neurointerventional approach for pseudoaneurysm treatment. The use of Onyx 18 provides optimism for the success of minimally invasive approaches to pseudoaneurysm treatment in the head and neck area without the need for coiling. 
